# Biodetoxification of Lignocellulose Hydrolysate for Direct Use in Succinic Acid Production

**DOI:** 10.34133/bdr.0044

**Published:** 2024-08-15

**Authors:** Wankui Jiang, Zhixiao Lei, Haiyan Gao, Yujia Jiang, Carol Sze Ki Lin, Wenming Zhang, Fengxue Xin, Min Jiang

**Affiliations:** ^1^ State Key Laboratory of Materials-Oriented Chemical Engineering, College of Biotechnology and Pharmaceutical Engineering, Nanjing Tech University, Nanjing 211816, P.R. China.; ^2^ Jiangsu National Synergetic Innovation Center for Advanced Materials (SICAM), Nanjing Tech University, Nanjing 211816, P.R. China.; ^3^ School of Energy and Environment, City University of Hong Kong, 999077 Hong Kong, P.R. China.

## Abstract

The pretreatment of lignocellulosic biomass with acid generates phenolic and furanyl compounds that function as toxins by inhibiting microbial growth and metabolism. Therefore, it is necessary to detoxify acid-pretreated lignocellulosic biomass for better utilization. Among the various detoxification methods that are available, biodetoxification offers advantages that include mild reaction conditions and low energy consumption. In this study, a newly isolated *Rhodococcus aetherivorans* strain, N1, was found to effectively degrade various lignin-derived aromatic compounds, such as *p*-coumarate, ferulate, syringaldehyde, furfural, and 5-hydroxymethylfurfural. Furthermore, the metabolic pathway and genes responsible for this degradation were also identified. In addition, the overexpression of a demethylase (DesA) and 3,4-dioxygenase (DesZ) in strain N1 generated a recombinant strain, N1-S, which showed an enhanced ability to degrade syringaldehyde and 80.5% furfural, 50.7% 5-hydroxymethylfurfural, and 71.5% phenolic compounds in corn cob hydrolysate. The resulting detoxified hydrolysate was used directly as a feedstock for succinate production by *Escherichia coli* suc260. This afforded 35.3 g/l succinate, which was 6.5 times greater than the concentration afforded when nondetoxified hydrolysate was used. Overall, the results of this study demonstrate that strain N1-S is a valuable microbe for the biodetoxification of lignocellulosic biomass.

## Introduction

Lignocellulose consists of cellulose, hemicellulose, and lignin and is the most abundant and renewable resource on Earth. Thus, lignocellulosic biomass has been recognized as a potential raw material for the production of biofuels and biochemicals, such as ethanol, succinic acid, and lactic acid [1–5]. However, the crystalline structure of lignocellulosic biomass means that it is biorecalcitrant, which hinders its direct utilization. Generally, this is overcome by pretreatment of lignocellulosic biomass to release reducing sugars [6]. Various pretreatment methods are used, such as chemical methods (dilute acid, alkaline, or alkaline-oxidation treatments), physical methods (high-temperature pyrolysis, microwave irradiation, or crushing), and biological methods [7–10]. These pretreatments generate various compounds, such as lignin-derived aromatic compounds (e.g., vanillin, *p*-hydroxybenzoate, and ferulate), furanyl aldehydes (furfural and 5-hydroxymethylfurfural), and low-molecular-weight organic acids (acetic acid [CH_3_CO_2_H] and formic acid [HCO_2_H]) [11–13]. These compounds are toxic to microorganisms at certain concentrations, as they severely inhibit their growth and activity, hindering the industrial production of biofuels and biochemicals from pretreated lignocellulosic biomass [14–16]. For example, 1.4 mM coniferyl aldehyde has been found to completely inhibit the growth of *Saccharomyces cerevisiae* [17,18]. Therefore, pretreated lignocellulosic biomass must be detoxified to remove the abovementioned compounds and thereby improve its fermentability.

The detoxification of pretreated lignocellulosic biomass can be achieved via different methods, such as water (H_2_O) washing, evaporation, organic liquid–liquid extraction, ion-exchange adsorption, and activated carbon adsorption [14–16,19–23]. However, these methods have several disadvantages, such as their extensive use of freshwater and generation of wastewater, high energy consumption, and incomplete removal of inhibitors [24]. An alternative method that is attracting much attention is biodetoxification, in which pretreated lignocellulosic biomass is treated with microorganisms or their enzymes to remove toxic compounds or convert them into less toxic compounds [25]. Compared with other detoxification methods, biodetoxification has several advantages, such as mild reaction conditions, low consumption of fresh water, minimal wastewater production, and low energy consumption [14,26]. There have been several reports on the use of microorganisms for the detoxification of pretreated lignocellulosic biomass, i.e., lignocellulosic hydrolysates. For example, *Kurthia huakuii* LAM0618 was found to be capable of degrading phenolic compounds in lignocellulosic hydrolysate. In addition, using the detoxified hydrolysate directly as a feedstock for the production of lactic acid afforded a production of 33.47 g/l, which was 1.87 times greater than that obtained from nondetoxified hydrolysate [27]. Moreover, *Issatchenkia occidentalis* CCTCC M206097 removed 67% of syringaldehyde, 73% of ferulate, 62% of furfural, and 85% of 5-hydroxymethylfurfural from pretreated lignocellulosic hydrolysate over 24 h [28]. Similarly, *Coniochaeta ligniaria* NRRL30616 removed more than 95% of the acetate and 65% of the furfural, hydroxymethylfurfural, and phenolic compounds generated in corn stover during pretreatment [21]. Furthermore, *Aspergillus oryzae* ZN1 was reported to degrade furfural and 5-hydroxymethylfurfural produced during the pretreatment of corn straw with dilute acid. In addition, the use of the detoxified hydrolysate as a feedstock for lactic acid production afforded a production of 104.5 g/l [29].

However, although the abovementioned microorganisms have been employed for biological detoxification, few microorganisms can simultaneously degrade lignin-derived phenolic and furanyl compounds, and their detoxification mechanisms have not been elucidated. At present, some studies on the microbial metabolism of lignin-derived phenolic compounds have been reported to provide guidance in the study of microbial detoxification mechanisms. For example, Wierckx et al. [30] comprehensively summarized the metabolic pathways and enzymes involved in the microbial metabolism of lignin-derived phenolic compounds, providing a foundation for the microbial detoxification of lignin-derived phenolic compounds. Lubbers et al. [31] reported that microbial metabolism of furanyl aldehydes occurs mainly through oxidation and/or reduction to the functional alcohol and acid forms and summarized the relevant metabolic pathways. In this study, a newly isolated *Rhodococcus aetherivorans* strain, N1, was found to degrade lignin; thus, its ability to degrade lignin-derived phenolic compounds and furanyl aldehydes was explored. Furthermore, genetic modification of strain N1 yielded a recombinant strain, N1-S, which exhibited an enhanced ability to degrade phenolic compounds and furanyl aldehydes. Moreover, strain N1-S was used for the biological detoxification of corn cob hydrolysate obtained from the pretreatment of lignocellulosic biomass with dilute acid. When the resulting detoxified hydrolysate was used directly as a feedstock for *Escherichia coli* suc260, the production of succinic acid reached 35.3 g/l, which was 6.5 times greater than that when nondetoxified hydrolysate was used.

## Materials and Methods

### Strains and growth conditions

Seed cultures of *R. aetherivorans* N1 (China Center for Type Culture Collection no. M20221270, GenBank accession no. P106982-CP106084) and *E. coli* suc260 (China Center for Type Culture Collection, no. M2012351) in Luria–Bertani (LB) broth containing 10.0 g/l tryptone, 5.0 g/l yeast extract, and 10.0 g/l sodium chloride (NaCl) were prepared and then incubated at 30°C and 37°C with shaking at 180 rpm [32]. Phenolic compounds and furanyl aldehydes were degraded using a mineral salt medium (MSM, pH 7.0) that contained 1.0 g/l ammonium nitrate, 1.0 g/l NaCl, 1.5 g/l dipotassium phosphate, 0.5 g/l monopotassium phosphate, and 0.2 g/l magnesium sulfate heptahydrate (MgSO_4_·7H_2_O). M10 medium was prepared by adding 10 g/l glucose and 2.0 g/l urea to MSM. The fermentation medium for *E. coli* suc260 contained corn cob hydrolysate, 0.12 g/l betaine, 2.6 g/l diammonium phosphate, 0.87 g/l monoammonium phosphate, 0.14 g/l potassium chloride, and 0.37 g/l MgSO_4_·7H_2_O, together with trace compounds (as reported by Dong et al.) [1].

### Degradation of lignocellulose-derived phenolic compounds and furanyl aldehydes

The degradation of lignocellulose-derived phenolic compounds and furanyl aldehydes by *R. aetherivorans* strains was investigated using various representative substrates, i.e., phenolic compounds and furanyl aldehydes, namely, *p*-hydroxybenzoate, *p*-coumarate, ferulate, vanillin, coniferyl alcohol, syringaldehyde, sinapate, furfural, and 5-hydroxymethylfurfural. M10 medium containing 100 mg/l phenolic compounds and furanyl aldehydes was inoculated with 10% v/v strain N1 seed culture and then divided into several cultures. The cultures were incubated at 30°C with shaking at 180 rpm. After 24 h, the concentrations of the phenolic compounds and furanyl aldehydes were determined. To further investigate the degradation of 5 of the abovementioned lignocellulose-derived phenolic compounds and furanyl aldehydes, MSM containing 300 mg/l syringaldehyde, ferulate, *p*-coumarate, furfural, or 5-hydroxymethylfurfural were inoculated with 2% v/v strain N1 seed culture and then divided into several cultures. These cultures were then incubated at 30°C with shaking at 180 rpm for 196 h. During this period, samples were taken every 24 h and analyzed to determine bacterial growth and substrate degradation.

To examine whether the concentrations of phenolic compounds and furanyl aldehydes affected their degradation by strain N1, MSM supplemented with various concentrations (100, 200, 300, 400, and 500 mg/l) of ferulate, *p*-coumarate, furfural, and 5-hydroxymethylfurfural were inoculated with 20% v/v strain N1 seed culture. The resulting cultures were incubated for 24 h, with samples taken every 2 h and analyzed to determine the degradation of substrates.

To examine whether mixtures of phenolic compounds and furanyl aldehydes could be degraded by strain N1, aliquots of MSM containing different concentrations (1, 2, 4, 6, and 8 g/l) of a mixture of phenolic compounds (furfural:5-hydroxymethylfurfural:*p*-hydroxybenzoate:vanillin:syringaldehyde:ferulate:*p*-coumarate = 271:505:16:68:50:102:147) were inoculated with 2% v/v N1 seed culture. The resulting cultures were incubated for 288 h. During this period, samples were taken every 24 h and analyzed to determine cell growth and the degradation of phenolic compounds.

### Construction of a recombinant *R. aetherivorans* strain with an enhanced ability to degrade phenolic compounds

Detailed information on the primers and genes used to construct the expression system is provided in the Supplementary Materials. Briefly, a demethylase (DesA) and 3-*O*-methylgallate 3,4-dioxygenase (DesZ) from *Sphingobium* sp. SYK-6 were overexpressed in strain N1 [31]. The plasmid pNV18.1 was used for protein expression, and the construction method was based on previous reports [33,34]. The constructed vector pNV-AZ utilized the *plac* promoter carried by pNV18.1 itself as the promoter for *desA*, whereas *pami* was the promoter for *desZ* [35]. To verify the transcription of exogenous genes, the RNA of strain N1-S was extracted, and cDNA was obtained through reverse transcription. PCR amplification was performed using the primers desA-F/R and desZ-F/R. The methods for RNA extraction and reverse transcription followed those of Ke et al. [36].

### Preparation of dilute-acid hydrolysate of corn cobs

Corn cobs were crushed, and the resulting material was screened using a 40-mesh sieve. The screened solids were pretreated with 3% sulfuric acid (H_2_SO_4_) at a solid-to-liquid ratio of 1:7.5 (m:V), and the resulting mixture was heated at 126°C for 2.5 h. The mixture was subsequently filtered to obtain a dilute acid hydrolysate of corn cobs. The total concentration of reducing sugars in the hydrolysate was approximately 36 g/l, and its main components were glucose, xylose, and arabinose at a ratio of 6:32:5.

### Detoxification and subsequent fermentation of corn cob hydrolysate to produce succinic acid

Detoxification: The corn cob hydrolysate was adjusted to pH 7.0 by the addition of 5 M sodium hydroxide solution and then sterilized at 121°C for 20 min. The sterilized hydrolysate was inoculated with 20% v/v strain N1 inoculum, and the resulting culture was incubated at 30°C with shaking at 180 rpm for 168 h. During this period, samples were taken every 24 h and analyzed to determine cell growth, the concentration of reducing sugars, and the total concentrations of soluble phenolic compounds and furanyl aldehydes.

Fermentation: Detoxified corn cob hydrolysate was centrifuged at 12,000 rpm and then filtered through a membrane with a pore size of 0.22 μm. The resulting filtrate was used as the fermentation medium for *E. coli* suc260 instead of water. Specifically, 27 ml of filtrate was added to anaerobic bottles, and the loaded bottles were flushed with carbon dioxide for 4 min and then sterilized at 121°C for 20 min. Each bottle was subsequently inoculated using a sterile syringe with 3 ml of an *E. coli* suc260 seed mixture and then incubated at 37°C with shaking at 180 rpm for 72 h. During this period, samples were taken every 12 h and analyzed to determine the consumption of reducing sugars and the production of succinic acid. During fed-batch fermentation, 30 g/l glucose was added after 60 h of fermentation.

### Analytical methods

The cell mass was determined by measuring the optical density at 600 nm (OD_600_) by ultraviolet–visible spectrophotometry. The concentrations of phenolic compounds and furanyl aldehydes were determined using a high-performance liquid chromatography (HPLC) system (Dionex UltiMate 3000, Thermo Fisher Scientific, USA) equipped with a C_18_ reverse-phase column (5 μm, 4.6 mm × 250 mm), which was heated to 40°C and eluted with CH_3_OH:H_2_O:CH_3_CO_2_H (70:24:1, v:v:v), where CH_3_OH is methanol, at a flow rate of 1.0 ml/min. The detection wavelength was 230 nm, and the sample volume was 20 μl. The concentrations of reducing sugars in the corn cob hydrolysate were determined using the dinitrosalicylic acid method [37]. The concentrations of glucose, xylose, and succinic acid were determined by HPLC using an ion-exchange chromatography column (Aminex HPX-87H, 300 mm, Bio-Rad, USA) heated to 55°C and an RI101 refractive index detector (Shodex, USA). The column was eluted with 5 mM H_2_SO_4_ at a flow rate of 0.6 ml/min, and the sample volume was 20 μl. The total concentrations of phenolic compounds in the corn cob dilute-acid hydrolysate were determined using the Folin–Ciocalteu method [27]. The concentrations of phenolic compounds and furanyl aldehydes in the hydrolysate were determined by HPLC. The column was heated to 30°C and eluted with a gradient of mobile phase A (0.02 mol/l monosodium phosphate containing 5% acetonitrile [CH_3_CN] adjusted to pH 2.9 by the addition of phosphoric acid) and mobile phase B (acetonitrile/methanol = 1:1 [v:v]), which consisted of 100% mobile phase A for 0 to 13.8 min and 66% mobile phase A and 34% mobile phase B for 13.8 to 45.0 min at a flow rate of 1 ml/min. The detection wavelength was 270 nm.

## Results and Discussion

### Degradation of lignocellulose-derived phenolic compounds and furanyl aldehydes by N1, a newly isolated strain of *R. aetherivorans*

**Fig. 1.**
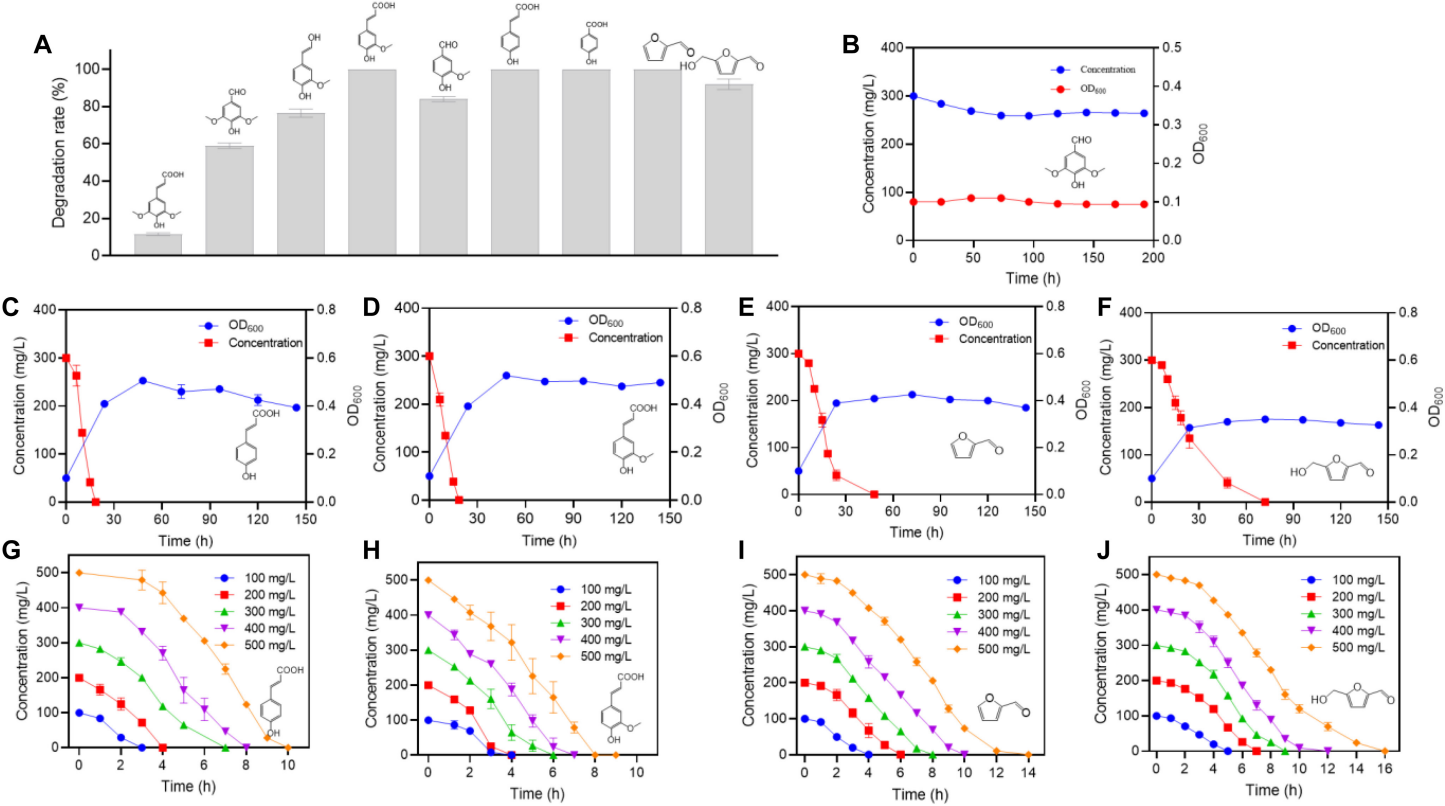
Degradation of lignin-derived aromatic compounds by *Rhodococcus aetherivorans* N1. (A) Rates of degradation of 7 lignin-derived aromatic hydrocarbons (left to right: sinapate, syringaldehyde, coniferyl alcohol, ferulate, *p*-hydroxybenzoate, *p*-coumarate, furfural, and 5-hydroxymethylfurfural) by strain N1. (B to F) Time course of the utilization of syringaldehyde, ferulate, *p*-coumarate, furfural, and 5-hydroxymethylfurfural, respectively, as the sole carbon source for growth by strain N1. (G to J) Time courses of the degradation of different concentrations of *p*-coumarate, ferulate, furfural, and 5-hydroxymethylfurfural by strain N1.

Previously, a newly isolated *R. aetherivorans* strain, N1, was found to be able to survive in culture with lignin as the sole carbon source [32]. To further investigate the ability of strain N1 to degrade lignin-derived aromatic compounds and furanyl aldehydes, its abilities to degrade *p*-hydroxybenzoate, *p*-coumarate, ferulate, vanillin, coniferyl alcohol, syringaldehyde, sinapate, furfural, and 5-hydroxymethylfurfural were examined. As shown in Fig. 1A, strain N1 completely degraded *p*-hydroxybenzoate, *p*-coumarate, ferulate, and furfural over 24 h. Moreover, strain N1 partially degraded vanillin, coniferyl alcohol, syringaldehyde, and 5-hydroxymethylfurfural, with degradation percentages ranging from 59% to 84%, respectively, over 24 h. However, strain N1 had a weak ability to degrade sinapate, as evidenced by sinapate being degraded by only 11% over 24 h. Previous studies have established that *Pseudomonas putida* KT2440 can degrade various phenolic compounds, such as vanillin, vanillate, *p*-hydroxybenzoate, *p*-coumarate, and ferulate, derived from guaiacyl-type (G-type) and *p*-hydroxyphenyl-type (H-type) lignins. However, few studies have reported that *P. putida* KT2440 can degrade syringaldehyde and other phenolic compounds derived from syringyl-type (S-type) lignins [31,38]. In the present study, unlike *P. putida* KT2440, strain N1 not only degraded G- and H-type lignin-derived aromatic compounds but also partially degraded S-type lignin-derived aromatic compounds such as sinapate and syringaldehyde. Therefore, strain N1 has greater potential than *P. putida* KT2440 for application in the degradation of lignin-derived phenolic compounds and furanyl aldehydes.

To further investigate the ability of strain N1 to degrade lignin-derived aromatic compounds, its abilities to degrade 3 representative phenolic compounds, namely, *p*-coumarate (derived from H-type lignin), ferulate (derived from G-type lignin), and syringaldehyde (derived from S-type lignin), and 2 representative furanyl aldehydes, namely, furfural and 5-hydroxymethylfurfural, were examined. As depicted in Fig. 1C to F, strain N1 was capable of utilizing *p*-coumarate, ferulate, furfural, or 5-hydroxymethylfurfural as its sole carbon source for growth, as it completely degraded 300 mg/l *p*-coumarate or ferulate within 18.5 h and 300 mg/l furfural or 5-hydroxymethylfurfural within 72 h. However, strain N1 only partially degraded syringaldehyde, i.e., by only 12.0%, within 200 h (Fig. 1B). Thus, strain N1 was unable to grow when syringaldehyde was its sole carbon source. Furthermore, strain N1 degraded different concentrations of *p*-coumarate, ferulate, furfural, and 5-hydroxymethylfurfural without exhibiting high-concentration inhibition or induction effects (Fig. 1G to J). For example, when a medium was inoculated with 20% v/v of a seed culture of strain N1, the resulting culture completely degraded 200 mg/l *p*-coumarate or ferulate within 4 h. Similarly, when the medium was inoculated with 20% v/v seed culture of strain N1, the resulting culture completely degraded 500 mg/l ferulate within 8 h and 500 mg/l *p*-coumarate within 10 h (Fig. 1G and H). Moreover, strain N1 degraded 100 mg/l furfural and 5-hydroxymethylfurfural within 4 to 6 h and 500 mg/l furfural and 5-hydroxymethylfurfural within only 14 to 16 h (Fig. 1I and J). Taken together, these results indicate that strain N1 has strong abilities to degrade lignin-derived phenolic compounds (derived from G-type and H-type lignins) and furanyl aldehydes. Moreover, strain N1 rapidly degraded high concentrations of *p*-coumarate, ferulate, furfural, and 5-hydroxymethylfurfural. However, its ability to degrade S-type lignin-derived compounds such as syringaldehyde requires improvement.

### Analysis of the metabolic pathway responsible for the degradation of lignin-derived aromatic compounds in strain N1

**Fig. 2.**
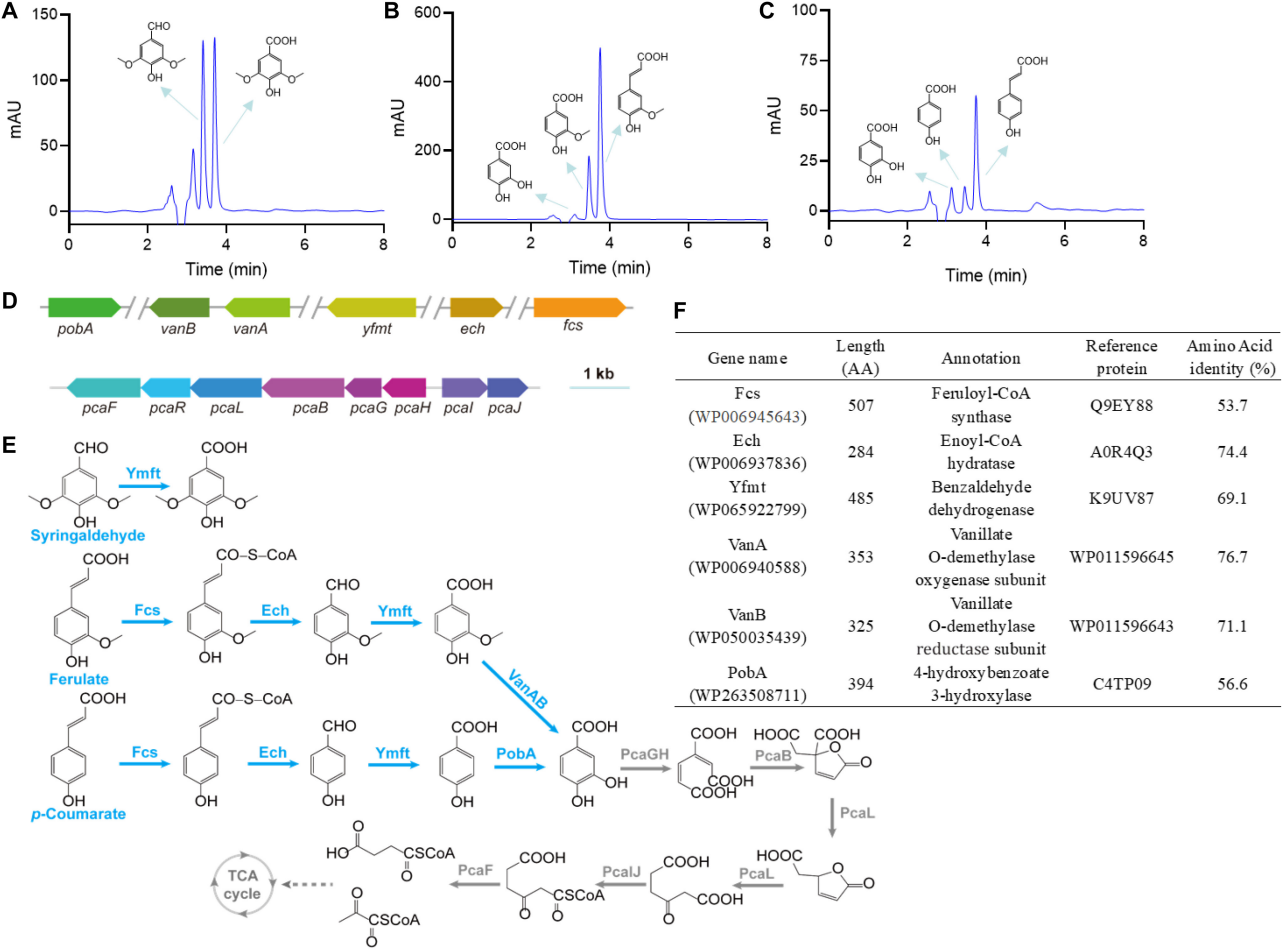
Degradation of syringaldehyde, ferulate, and *p*-coumarate in *R. aetherivorans* N1. (A to C) HPLC analyses of metabolites formed during the degradation of syringaldehyde, ferulate, and *p*-coumarate, respectively, by strain N1. (D) Gene cluster encoding proteins responsible for the degradation of syringaldehyde, ferulate, and *p*-coumarate in strain N1. (E) Degradation pathway of syringaldehyde, ferulate, and *p*-coumarate in strain N1. (F) Details of some of the genes encoding proteins involved in the degradation of syringaldehyde, ferulate, and *p*-coumarate in strain N1.

To further elucidate the mechanism whereby strain N1 degraded lignin-derived aromatic compounds, the metabolic pathway responsible for its degradation of 3 representative compounds, namely, *p*-coumarate, ferulate, and syringaldehyde, was analyzed. As shown in Fig. 2A, in a sample from a strain N1 culture in which syringaldehyde was the substrate, the compound with a retention time of 3.69 min was syringaldehyde, and the compound with a retention time of 3.39 min was syringate. Analogously, as shown in Fig. 2B, in a sample from a strain N1 culture in which ferulate was the substrate, 3 substances were detected, namely, ferulate, vanillate, and protocatechuate. Similarly, as shown in Fig. 2C, in a sample from a strain N1 culture in which *p*-coumarate was the substrate, 3 substances were detected, namely, *p*-coumarate, *p*-hydroxybenzoate, and protocatechuate.

Previous studies have reported the degradation pathways for *p*-coumarate, ferulate, and syringaldehyde [31]. In prokaryotic microorganisms, including *Sphingobium* sp. SYK-6 and *R. jostii* RHA1, the common metabolic pathway involves coenzyme A (CoA) synthase-catalyzed conversion of ferulate and *p*-coumarate to their corresponding CoA products, which are then converted by CoA hydratase into vanillin and *p*-hydroxybenzaldehyde, respectively [39,40]. A dehydrogenase subsequently converts vanillin and *p*-hydroxybenzaldehyde into vanillate and *p*-hydroxybenzoate, respectively. Vanillate is further converted into protocatechuate by a demethylase, and *p*-hydroxybenzoate can be converted to protocatechuate by a hydroxylase. Finally, protocatechuate is subjected to double oxygenase-catalyzed ring opening to yield carbon-compound building blocks. Comparative analysis of the genomes of strains N1, PD630 (GenBank accession no. GCA020542785.1), and RHA1 (GenBank accession no. GCA000014565.1), combined with sequence alignment in the NCBI database, revealed several related degradation genes, including *Fcs*, which encodes *trans*-ferulyl CoA synthase; *Ech*, which encodes enoyl CoA hydratase; *Yfmt*, which encodes a vanillin dehydrogenase; *VanAB*, which encodes a 2-component vanillate *O*-demethylase; and *PobA*, which encodes *p*-hydroxybenzoate hydroxylase (Fig. 2D to F). The vanillin dehydrogenase encoded by *Yfmt* degrades not only vanillin and *p*-hydroxybenzaldehyde but also syringaldehyde [41]. Based on the aforementioned findings, the metabolic pathways and genes of strain N1 that degrade ferulate, *p*-coumarate, and syringaldehyde were proposed. As shown in Fig. 2E, strain N1 completely degraded ferulate and *p*-coumarate, as verified by microbial growth and metabolite detection experiments in which each of the aforementioned compounds was the sole carbon source. However, strain N1 could degrade only syringaldehyde to syringate, indicating that its growth and metabolism were inhibited by the accumulation of syringate. Taken together, the abovementioned results revealed that strain N1 can degrade *p*-coumarate (derived from H-type lignins), ferulate (derived from G-type lignins), and syringaldehyde (derived from S-type lignins).

### Enhancement of syringaldehyde degradation by the recombinant *R. aetherivorans* strain N1-S

**Fig. 3.**
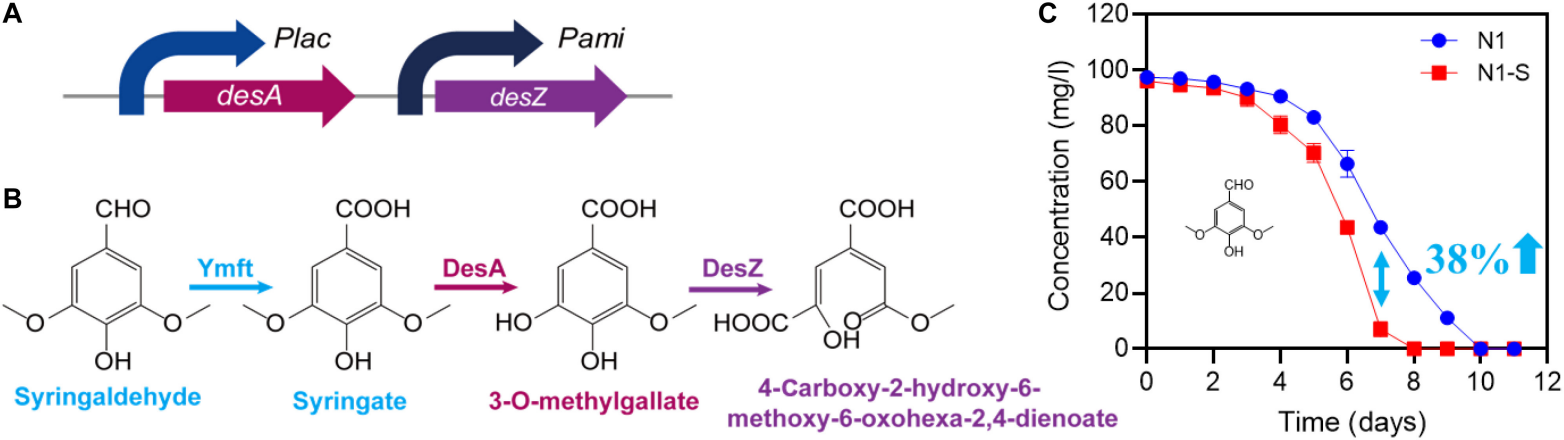
Generation of a recombinant *R. aetherivorans* strain N1-S, which has an increased ability to degrade syringaldehyde, and analysis of the degradative performances of both strains. (A) Construction of an expression vector for generating a recombinant strain from strain N1 that exhibited an increased ability to degrade syringaldehyde. (B) Metabolic pathway by which the recombinant strain *R. aetherivorans* N1-S degrades syringaldehyde. (C) Time courses of the degradation of syringaldehyde by strains N1 and N1-S.

The above-described results indicate that strain N1 only partially degraded syringaldehyde, i.e., to syringate, owing to a lack of genes encoding other syringate-degrading enzymes. As mentioned, the resulting accumulation of syringate inhibited the growth of strain N1. Thus, genes encoding enzymes involved in the degradation of syringate conversion were overexpressed in strain N1, as described below. *Sphingobium* sp. SYK-6 was previously reported to possess an aldehyde dehydrogenase (DesV) that enables it to degrade syringaldehyde to syringate [31]. Syringate can then be converted by a demethylase (DesA) to 3-*O*-methylgallate, which is further metabolized by 3-*O*-methylgallate 3,4-dioxygenase (DesZ) to 4-carboxy-2-hydroxy-6-methoxy-6-oxohexa-2,4-dienoate [42]. Accordingly, *desA* and *desZ* from the genome of *Sphingobium* sp. SYK-6 were synthesized. Subsequently, plasmids containing *desA* and *desZ* were constructed and then transformed into strain N1 to afford a recombinant strain with an increased ability to degrade syringaldehyde (Fig. 3A). As shown in Fig. 3B, the recombinant *R. aetherivorans* N1 strain, N1-S, was able to metabolize 3-*O*-methylgallate to 4-carboxy-2-hydroxy-6-methoxy-6-oxohexa-2,4-dienoate, reducing the feedback inhibition caused by syringate accumulation during the degradation of syringaldehyde. 4-Carboxy-2-hydroxy-6-methoxy-6-oxohexa-2,4-dienoate was not detected, suggesting that it might have been further metabolized by the recombinant strain N1-S. Moreover, under similar conditions, the recombinant strain N1-S showed a 38% greater ability to degrade syringaldehyde than the wild-type strain, i.e., N1 (Fig. 3C). This result demonstrated that the recombinant strain N1-S is more effective than strain N1 in detoxifying lignocellulose hydrolysates by degrading their phenolic compounds.

### Recombinant strain N1-S-based detoxification of a hydrolysate obtained by acid pretreatment of corn cobs

**Fig. 4.**
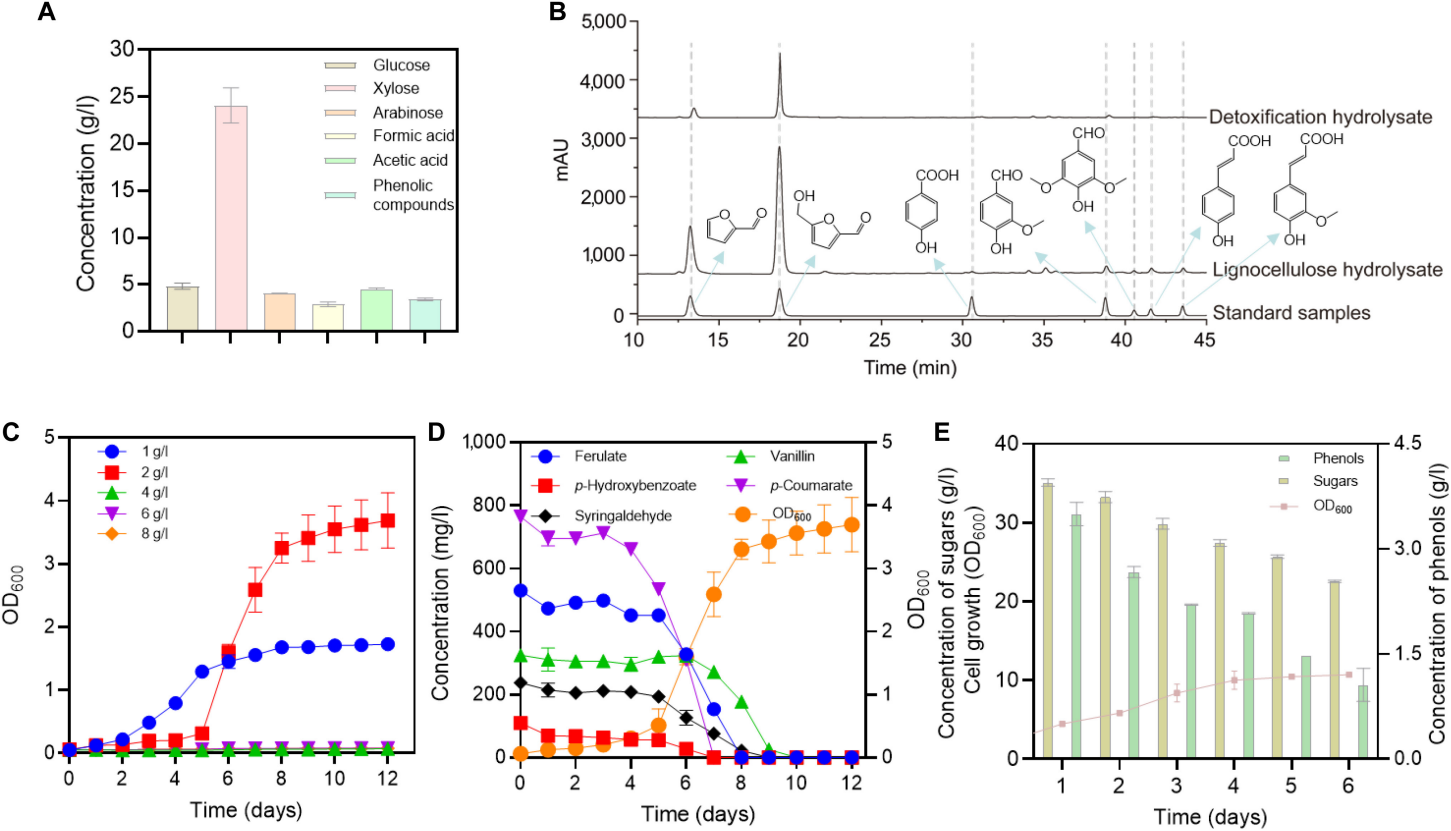
Details of the corn cob hydrolysate and its detoxification. (A) Composition of corn cob hydrolysate. (B) High-performance liquid chromatograms of inhibitors in nondetoxified and detoxified corn cob hydrolysates. (C) Time course of the use of a mixed phenolic system (furfural:5-hydroxymethylfurfural:*p*-hydroxybenzoate:vanillin:syringaldehyde:ferulate:*p*-coumarate = 271:505:68:50:102:147, based on the composition and proportion of phenolic compounds detected in the hydrolysate) as the sole carbon source for growth by the recombinant strain N1-S. (D) Time course of the degradation of 2 g/l mixed phenolic compounds by strain N1-S. (E) Time course of the detoxification of corn cob hydrolysate by the recombinant strain N1-S.

To further test the degradation ability of the recombinant strain N1-S, it was used to degrade corn cob hydrolysate, and the products were analyzed. A compositional analysis of the hydrolysate, which was obtained by pretreatment of corn cobs with dilute acid, is shown in Fig. 4A, revealing that it contained 36 g/l of reducing sugars, including 24 g/l of xylose, 4.8 g/l of glucose, and 4.0 g/l of arabinose; had a total phenolic compound concentration of 3.7 g/l; contained organic acids, namely, 2.9 g/l of HCO_2_H and 4.5 g/l of CH_3_CO_2_H; and contained 776.2 mg/l of furanyl aldehydes, including 270.9 mg/l of furfural and 505.3 mg/l of 5-hydroxymethylfurfural. Furthermore, HPLC analysis of the total concentrations of phenolic compounds and furanyl aldehydes in the hydrolysate is shown in Fig. 4B. Furfural, 5-hydroxymethylfurfural, *p*-hydroxybenzoate, vanillin, syringaldehyde, *p*-coumarate, and ferulate were detected at concentrations of 270.9, 505.3, 16.4, 68.1, 50.4, 147.3, and 102.2 mg/l, respectively. The total concentrations of monomeric phenolic compounds determined by HPLC were much lower than the total concentrations of soluble phenolic compounds in the hydrolysate, which may indicate that phenolic compounds exist as monomers and oligomers, as the latter cannot be detected by HPLC. To further investigate the ability of the recombinant strain N1-S to degrade mixtures of phenolic compounds and furanyl aldehydes, different concentrations of a specific mixture (furfural:5-hydroxymethylfurfural:*p*-hydroxybenzoate:vanillin:syringaldehyde:ferulate:*p*-coumarate = 271:505:16:68:50:102:147, based on the composition of the hydrolysate mentioned above) were used as substrates. As shown in Fig. 4C, the recombinant strain N1-S was able to grow in a culture containing 2 g/l of this mixture, but its growth was inhibited in a culture containing more than 4 g/l of this mixture. Within 144 h, furfural, 5-hydroxymethylfurfural, ferulate, *p*-hydroxybenzoate, syringaldehyde, and *p*-coumarate were degraded by 63%, 49%, 38.2%, 74.6%, 59.4%, and 47.0%, respectively (Fig. 4D). In contrast, vanillin was degraded by only 0.5%, but it was degraded significantly more after 144 h (Fig. 4D). Within 240 h of culture, all the components of the mixture were completely degraded by the recombinant strain N1-S (Fig. 4D). In addition, the recombinant strain N1-S also tended to degrade *p*-hydroxybenzoate furfural and 5-hydroxymethylfurfural, followed by the degradation of *p*-coumarate, ferulate, syringaldehyde, and vanillin. Similarly, Cai et al. [35] studied the ability of *R. opacus* PD630 to degrade a mixture of phenolic compounds containing 140 mg/l vanillin, 190 mg/l ferulate, 170 mg/l vanillate, 140 mg/l *p*-hydroxybenzoate, 180 mg/l syringaldehyde, and 220 mg/l sinapate, to which 2 g/l glucose was added. They reported that *R. opacus* PD630 degraded all of the abovementioned components, except for sinapate, within 40 h [35]. Compared with *R. opacus* PD630, the recombinant strain N1-S was able to degrade and tolerate higher concentrations of phenolic compounds, indicating that it has greater potential for application in detoxifying lignocellulosic hydrolysates.

To further investigate the detoxification ability of the recombinant strain N1-S, it was inoculated into undetoxified corn cob hydrolysate. As shown in Fig. 4E, the rate of removal of phenolic compounds from the hydrolysate reached 45.8% after 96 h, accompanied by the consumption of 9.4 g/l reducing sugars (initial reducing sugar concentration of 36.8 g/l). After 144 h, the rate of removal of phenolic compounds from the hydrolysate reached 71.5%, accompanied by the consumption of 14.3 g/l reducing sugars. During this period, the OD_600_ of the recombinant strain N1-S increased from 2.1 to 10.7. HPLC analysis revealed that the recombinant strain N1-S completely degraded *p*-hydroxybenzoate, *p*-coumarate, ferulate, and syringate, leaving only 29% vanillin. In addition, the rates of degradation of furfural and 5-hydroxymethylfurfural reached 80.5% and 50.7%, respectively. These results indicate that the recombinant strain N1-S not only effectively degrades phenolic compounds but also degrades furanyl aldehydes, such as furfural and 5-hydroxymethylfurfural.

Lignocellulose detoxification has been reported to be performed by many microorganisms, including *K. huakuii*, *I. occidentalis*, *C. ligniaria*, *A. oryzae*, and *Ureibacillus thermosphaericus* [21,27–29,43]. For example, *U. thermosphaericus* degraded furfural and 5-hydroxymethylfurfural in a wood hydrolysate obtained by the pretreatment of wood with dilute acid, and the resulting detoxified hydrolysate was used for ethanol production by recombinant *E. coli*. However, *U. thermosphaericus* was found to be unable to degrade phenolic compounds [43]. *K. huakuii* was shown to convert syringaldehyde, hydroxybenzaldehyde, and vanillin present in corn straw hydrolysate into the less toxic acids that are used in lactic acid production. However, its ability to degrade furfural and 5-hydroxymethyl furfural has not been explored [27]. Thus, no study has reported strains that can simultaneously degrade all types of lignin-derived aromatic compounds, furfural, and 5-hydroxymethylfurfural. In contrast, the recombinant strain N1-S constructed in this study was able to degrade S-, G-, and H-type lignin-derived aromatic compounds, including furfural and 5-hydroxymethylfurfural. As such, compared with the abovementioned microbes, the recombinant strain N1-S has significant advantages in terms of the wide spectrum of substrates it can degrade and its tolerance of phenolic compounds.

### Production of succinic acid from detoxified corn cob hydrolysate by *E. coli* suc260

**Fig. 5.**
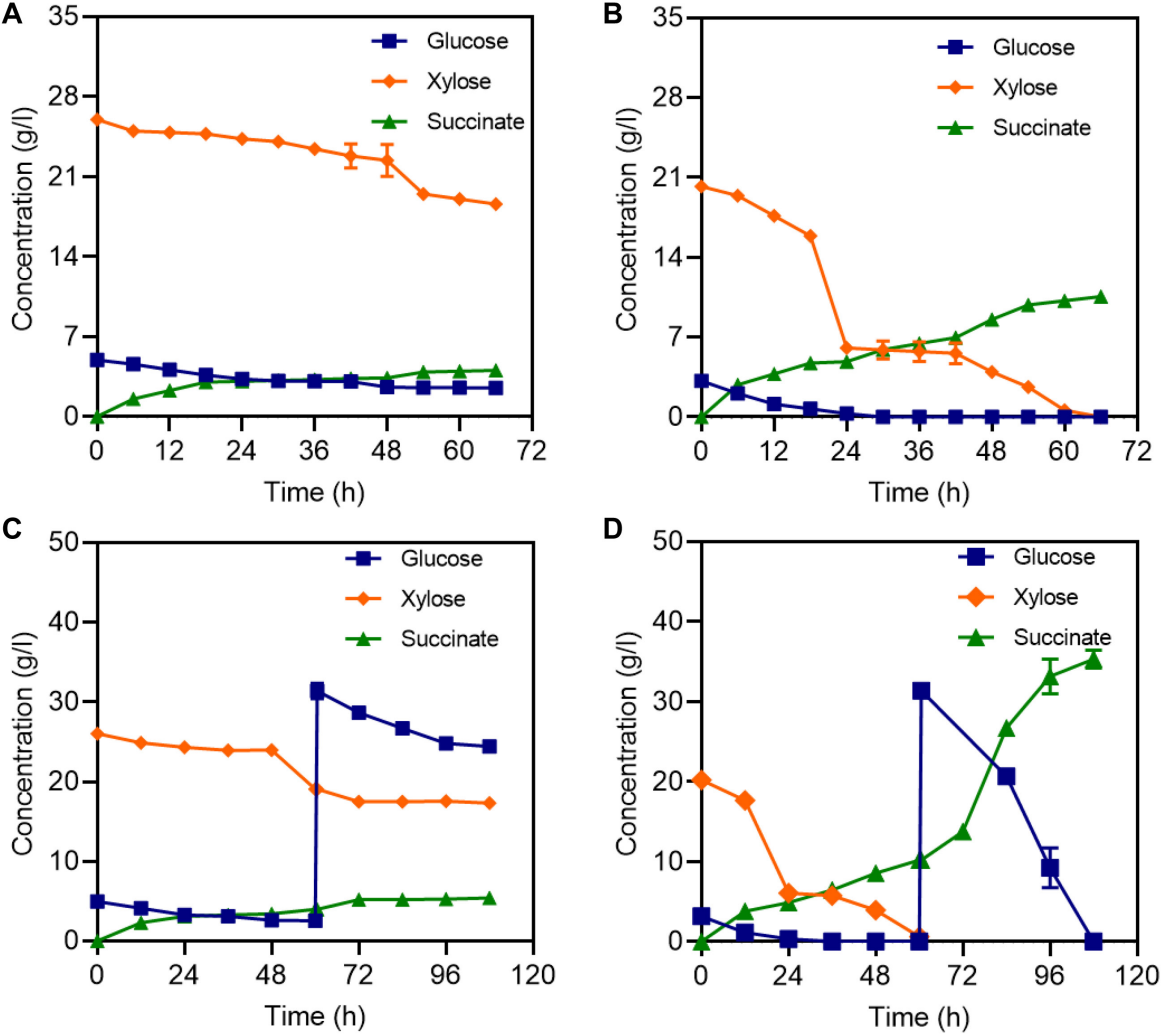
Fermentation of corn cob hydrolysate not detoxified and detoxified by the recombinant strain N1-S by *E. coli* suc260 to produce succinate, without and with the addition of glucose. (A and B) Fermentation of corn cob hydrolysate not detoxified and detoxified by the recombinant strain N1-S, respectively, by *E. coli* suc260 to produce succinate. (C and D) Fermentation of corn cob hydrolysate not detoxified and detoxified by the recombinant strain N1-S, respectively, with the addition of 30 g/l glucose after 60 h of fermentation.

To further explore whether corn cob hydrolysate detoxified by the recombinant strain N1-S was suitable for the direct production of chemicals, the detoxified hydrolysate was employed as a substrate for succinic acid production by *E. coli* suc260. The latter microbe was constructed in our laboratory by genetic modification and adaptive evolution to eliminate its ability to generate by-products. *E. coli* suc260 was used, as it has been previously shown that it can generate rather high concentrations of succinic acid, with simultaneous utilization of glucose and xylose, but cannot tolerate the presence of phenolic compounds and furanyl aldehydes [44,45]. Thus, anaerobic fermentation was conducted for 96 h with *E. coli* suc260 and with corn cob hydrolysate detoxified by the recombinant strain N1-S as the substrate. As shown in Fig. 5A, after 72 h, 30 g/l reducing sugars were consumed, and 10.5 g/l succinic acid was produced. In contrast, with undetoxified corn cob hydrolysate as the substrate, after 72 h, only 13 g/l reducing sugars were consumed, and only 4 g/l succinic acid was produced, as the growth of *E. coli* suc260 was inhibited. Moreover, with the continuous addition of 30 g/l glucose, the succinic acid yield from the detoxified hydrolysate increased to 35.3 g/l, which was 6.5 times greater than the yield from the nondetoxified hydrolysate, i.e., 5.4 g/l. These results indicate that phenolic compounds in the nondetoxified hydrolysate strongly inhibited the metabolism of the succinic acid-producing strain *E. coli* suc260. Furthermore, the results indicate that the recombinant strain N1-S effectively removed phenolic and furanyl compounds from the hydrolysate, thereby facilitating the metabolism of *E. coli* suc260 and resulting in the efficient synthesis of succinic acid from the hydrolysate.

## Conclusion

In this study, a newly isolated *R. aetherivorans* strain, N1, was found to be capable of degrading various lignin-derived aromatic compounds, and its degradation pathway and related genes were elucidated. Furthermore, a recombinant strain, *R. aetherivorans* N1-S, was constructed that exhibited increased syringaldehyde degradation ability. Moreover, strain N1-S was able to degrade 2 g/l lignin-derived aromatic compounds and thereby utilize them as sole carbon sources. In addition, strain N1-S degraded 50% to 80% of the phenolic compounds, furfural, and 5-hydroxymethylfurfural in a corn cob hydrolysate obtained from dilute acid pretreatment of lignocellulosic biomass. The resulting detoxified hydrolysate was used directly as a feedstock for succinic acid production in *E. coli* suc260, affording a succinic acid production of 35.3 g/l, which was 6.5 times greater than that obtained by using nondetoxified hydrolysate as a feedstock.

## Data Availability

The data from this study will be made available upon request.
