# Supplementary material for: Biodetoxification of Lignocellulose Hydrolysate for Direct Use in Succinic Acid Production
Source: Biodes Res. 2024 Aug 15;6:0044. doi: 10.34133/bdr.0044 (PMC11325090; doi:10.34133/bdr.0044)
Supplement: Supplementary 1 — Tables S1 and S2 Fig. S1 [file bdr.0044.f1.pdf]

Supporting Information

Biodetoxification of lignocellulose hydrolysate for direct use in succinic acid production

Wankui Jiang[1], Zhixiao Lei[1], Haiyan Gao[1], Yujia Jiang[1,2], Carol Sze Ki Lin[3], Wenming Zhang[1,2]*, Fengxue Xin[1,2]*, Min Jiang[1,2]

[1]State Key Laboratory of Materials-Oriented Chemical Engineering,

College of Biotechnology and Pharmaceutical Engineering,

Nanjing Tech University, Nanjing, 211816, P.R. China

[2]Jiangsu National Synergetic Innovation Center for Advanced Materials (SICAM), Nanjing Tech University, Nanjing, 211816, P.R. China

[3]School of Energy and Environment, CityUniversity of Hong Kong, 999077 Hong Kong, PR Ch

*Corresponding authors at: State Key Laboratory of Materials-Oriented Chemical Engineering, College of Biotechnology and Pharmaceutical Engineering, Nanjing Tech University, Puzhu South Road 30#, Nanjing 211816, P. R. China.

E-mail address: zhangwm@njtech.edu.cn (Wenming Zhang);

xinfengxue@njtech.edu.cn (Fengxue Xin)

Table S1 Strains and plasmids used in this study

Strain or plasmids	Characteristics[a]	Sources or reference
Strains		
Rhodococcus aetherivorans N1	Wild-type, able to degrade inhibitors in corn cob dilute acid hydrolysate	Jiang *et al*., 2023
Rhodococcus aetherivorans N1-S	Strain N1 derivate with heterologous expression of DesV and DesV	This study
Escherichia coli Suc260	Succinic acid producing strain	Dong *et al*., 2017
Escherichia coli DH5α	*F⁻ recA1 endA1 thi-1 hsrdR17 supE44 relA1 deoR △(lacZYA-argF) U169 φ 80lacZ △M15*	TaKaRa
Plasmids		
pNV18.1	*Rhodococcus* protein expression vector, Km^r	Liang *et al*., 2020
pNV-AZ	pNV18.1 harboring *desV* and *desV*, Km^r	This study

[a]Km^r, kanamycin resistant.

Table S2 Primers and gene fragments used in this study

Primer	Sequence (5´ to 3´)	Purpose
up-desA	GCTATGACATGATTACGAATTCATGGCCAAGTCCCTGCAGGACGTC	To amplify *desA* gene
do-desA	CCGGGTACCGAGCTCGAATTCTTAAGCCTTCTTGGTGCGC	
up-pami	AATTCGAGCTCGGTACCCGGGGATCCTCTAGACATATGTGCGGACGGCGG	To amplify *pami* promoter
do-pami	GACGATCTCCGCCATGGATCCGGATCCCTCCTTAGTGACTCGC	
up-desZ	GCGAGTCACTAAGGAGGGATCCGGATCCATGGCGGAGATCGTC	To amplify *desZ* gene
do-desZ	TGCCTGCAGGTCGACTCTAGATTAACGCCAGCACACGAAGC	
up-pnv	TCTAGAGTCGACCTGCAGGCATGC	To linearized plasmid pNV18.1
do-pnv	GAATTCGTAATCATGTCATAGCTGTTTCCTGTGTG	
desA-F	CACCGCCGAAGGCTACGTC	To amplify partial *desA* gene (~250bp)
desA-R	TGGCCCATGGCGTCCGTC	
desZ-F	TGCCAGGCCAGCCTGGATC	To amplify partial *desZ* gene (~250bp)
desZ-R	ACGGAATGTATCCCGCGAC	
desA	ATGGCCAAGTCCCTGCAGGACGTCCTGGACAACGCGGGCAACGCCGTGGATTTCCTGAGGAACCAGCAGACGGGGCCGAATGTCTACCCGGGTGTCCCGGCCGAGTACTCTAACTGGCGGAACGAACAGCGCGCGTGGGCGAAAACGGCGGTGCTGTTCAACCAATCGTACCACATGGTGGAGCTGATGGTGGAAGGACCCGACGCGTTCGCGTTCCTCAACTACCTCGGGATCAACTCGTTCAAGAACTTCGCCCCCGGCAAGGCGAAGCAGTGGGTGCCGGTCACCGCCGAAGGCTACGTCATTGGCGACGTGATCCTCTTCTACCTGGCAGAGAATCAGTTCAACTTGGTCGGCCGCGCCCCGGCGATCGAGTGGGCCGAGTTCCACGCCGCCACCGGGAAATGGAACGTCACCCTGACTCGCGACGAGCGGACGGCCCTACGTACCGACGGCGTCCGGCGGCACTACCGGTTCCAGCTGCAGGGCCCTAACGCGATGGCCATCCTGACGGACGCCATGGGCCAGACACCGCCCGACCTCAAGTTCTTCAACATGGCCGACATCCAGATCGCCGGCAAGACCGTCGGCGCGCTGCGCCACGGCATGGCTGGGCAGCCCGGATACGAACTCTACGGCCCCTGGGCCGACTACGAGG	Demethylase DesA coding gene

	CGGTCCATTCGGCGCTCGTTGCCGCAGGAAAG<br>AACCACGGGCTGGCCTTGGTCGGTGGGCGTG<br>CGTACTCCTCCAATACCCTCGAATCCGGCTGG<br>GTACCAAGTCCGTTCCCCGGCTATCTGTTCGG<br>CGAAGGAAGCGCCGACTTCCGCAAGTGGGCG<br>GGCGAGAACTCGTATGGGGCCAAGTGCTCGA<br>TCGGAGGATCGTACGTGCCCGAGAGCCTCGA<br>GGGTTACGGCCTCACCCCGTGGGACATCGGTT<br>ACGGAATCATCGTTAAGTTCGACCACGACTTC<br>ATCGGCAAGGAAGCGCTCGAGAAGATGGCGA<br>ACGAGCCGCACCTGGAGAAGGTGACGCTTGC<br>CCTCGACGACGAGGACATGCTGCGGGTGATG<br>TCGAGCTACTTTTCCGATAGCGGTCGAGCGAA<br>GTACTTCGAGTTCCCGAGCGCGGTGTACTCGA<br>TGCACCCCTACGACAGCGTCCTCGTCGATGGG<br>AAGCACGTCGGGGTGTCCACGTGGGTCGGCT<br>ACTCATCCAACGAGGGTAAGATGCTCACGTTG<br>GCGATGATCGACCCGAAGTACGCCAAACCCG<br>GCACCGAGGTGTCGCTGCTCTGGGGCGAACC<br>GAACGGCGGCACGTCCAAACCCACCGTCGAA<br>CCGCATGAGCAGACCGAGATCAAGGCAGTGG<br>TCGCGCCGGTGCCGTACTCGGCGGTGGCACGC<br>ACCGGTTATGCAGATTCGTGGCGCACCAAGA<br>AGGCTTAA	
desZ	ATGGCGGAGATCGTCCTCGGCATCGGCACCTC<br>CCACGGGCCGATGCTCGTCACGCAGACCGAG<br>CAGTGGCGCTCGCGGCTGGCCTTCGACCAATC<br>CGTGAATCACGCCTGGCGGGGCGGTTCGTGGT<br>CCTACGACCAACTCGTTGCCGAGCGGGCCGA<br>CCAGAACTTCGCGGCGCAGATCACGCCCGAA<br>GCGATGACGGCGCACAACGCTCGCTGCCAGG<br>CCAGCCTGGATCAGCTGGCGGAGATCTTCTCC<br>GAGGCGAAGATCGACGTGGCAGTGATCCTCG<br>GCAACGACCAGATGGAAATCTTCGACGAACG<br>CCTGGTCCCGGCGTTCTCGGTCTTCTACGGCG<br>ACACCATCACCAACTACGAGTTCCCGCCGGA<br>ACGGATGGCCGCCCTGCCCCCCGGGATCAAC<br>CTGAGTGTCGCGGGATACATTCCGTCGGGCGG<br>GGCAGAGTACGCCGGTCAGCCGGAGCTGGCG<br>CGTAGCATCATCGCCCAGGCGATGGCCGACG<br>AGTTCGACGTCGCAGCCATGAAGGCCCTCCCG<br>AAGCCCGAGACCCCGCATGCGTTCGGATTCGT<br>GTACCGGCGGATCATGCGAGACAACCCGGTG	3-O-methylgallate 3,4-dioxygenase DesZ coding gene

	CCCTCCGTGCCAGTGCTCGTCAACACGTTCTA<br>CCCGCCGAACCAGCCCACCGTCCGCAGGTGTT<br>ACGAGTTCGGCAAATCGGTCCTGCGCGGCATC<br>CAGGCGTGGGAGTCGGACGCCCGGGTGGCCG<br>TCTTGGCTTCAGGAGGTCTCACTCACTTCGTC<br>ATCGATGAGGAGATCGACCGCCTGTTTTTCCA<br>GGCCATGGAGGACCGCGACATTGCGCGGCTT<br>GCCGACCTCGGTGAGGCGATCTTCCAGGACG<br>GCACCAGCGAACTCAAGAACTGGATTCCGTT<br>GGCCGGCATGATGGCCGAACTCGGGCTGGAT<br>CACGAGATCCTGGACTACGTCCCGTGCTATCG<br>CAGCGAGGCCGGGACGGGCAACGCGATGGGC<br>TTCGTGTGCTGGCGTTAA	
pami	CATATGTGCGGACGGCGGATACGTCGTGGCTG<br>GAGAAGGTTGGACGGTCGTCATGATAGGCACC<br>TTTTCTCAACGTCTTTGAAGTGCTGGTACATCC<br>GCAGCGGGCGATGCTCAGAGAATACATGCTGC<br>CTAACGGAAGTAAAGATCCACGGAGGTGGAC<br>GTGCAAAGGAACGGACCCTGCCTATCGCTGTG<br>AACAGGTGAGATTACGGAGAACGGGGCTTGT<br>GGCCGTCCCTGTCGTGTGGTAAAATGTCCACA<br>ACGTTGCAGTTCATGCAATGTGGAACACTTCA<br>AGTCGGAAGCAAACGTCGGGTCATGAGCGCC<br>CGGCGAGTCACTAAGGAGGGATCC	*pami* promoter

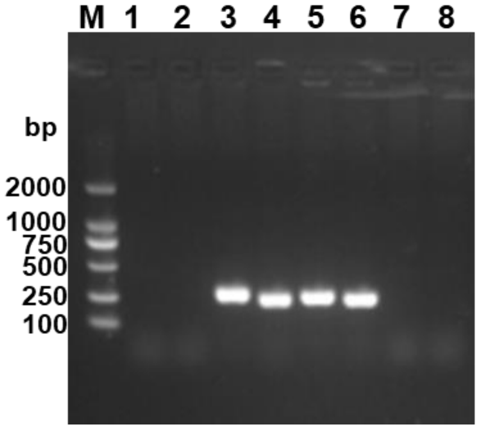

Fig. S1. PCR verification of transcription of exogenous genes in strain N1-S. Lanes: M molecular size markers; 1 and 2 products amplified with the primers of desA-F/R and desZ-F/R (template of RNA from strain N1-S); 3 and 4 products amplified with the primers of desA-F/R and desZ-F/R (template of cDNA from strain N1-S); 5 and 6 products amplified with the primers of desA-F/R and desZ-F/R (template of DNA from strain N1-S); 7 and 8 the negative controls amplified with the primers of desA-F/R and desZ-F/R (do not add templates).
